# Off-Label Uses of Anti-TNF Therapy in Three Frequent Disorders: Behçet's Disease, Sarcoidosis, and Noninfectious Uveitis

**DOI:** 10.1155/2013/286857

**Published:** 2013-08-01

**Authors:** Daniel Sánchez-Cano, José Luis Callejas-Rubio, Ricardo Ruiz-Villaverde, Raquel Ríos-Fernández, Norberto Ortego-Centeno

**Affiliations:** ^1^Internal Medicine Department, Hospital Santa Ana, 18600 Motril, Spain; ^2^Unidad de Enfermedades Autoinmunes Sistémicas, Hospital Universitario San Cecilio, 18012 Granada, Spain; ^3^Dermatology Department, Complejo Hospitalario Ciudad de Jaén, 23001 Jaén, Spain

## Abstract

Tumoral necrosis factor *α* plays a central role in both the inflammatory response and that of the immune system. Thus, its blockade with the so-called anti-TNF agents (infliximab, etanercept, adalimumab, certolizumab pegol, and golimumab) has turned into the most important tool in the management of a variety of disorders, such as rheumatoid arthritis, spondyloarthropatties, inflammatory bowel disease, and psoriasis. Nonetheless, theoretically, some other autoimmune disorders may benefit from these agents. Our aim is to review these off-label uses of anti-TNF blockers in three common conditions: Behçet's disease, sarcoidosis, and noninfectious uveitis. Due to the insufficient number of adequate clinical trials and consequently to their lower prevalence compared to other immune disorders, this review is mainly based on case reports and case series.

## 1. Introduction

Tumor necrosis factor- (TNF-) *α* is a pleiotropic cytokine which plays a major role in the development, homeostasis, and adaptive responses of the immune system. In fact, it is central to the initiation and maintenance of inflammation in multiple autoimmune and nonautoimmune disorders. TNF-*α*, which is released by macrophages, monocytes, and T lymphocytes, as well as other types of cells, can be found both in its soluble form and bound to the cellular membrane [1–3].

The better understanding of the pathogenesis of autoimmune disorders has led to the search of new targets for its treatment. In this way, biological therapies, which are synthesized from living organisms, can be designed to specifically act on certain inflammatory mediators, among them, TNF-*α*. Thus, reinforcement or even substitution of usual immunosuppressive therapies has now been made possible [1, 3].

Currently, five anti-TNF agents are commercially available: infliximab, etanercept, adalimumab, certolizumab pegol, and golimumab (Table 1). (a) Infliximab (INX) is an IgG1 chimeric monoclonal antibody with a constant human region and a variable murine one. This agent binds both the soluble and the cell-bound TNF-*α* but not TNF-*β*. It is administered intravenously on an outpatient basis, and even though serious infusion reactions are rare, development of antibodies against INX has been reported (incidence varying between 15% and 50%). The latter has been associated with a lower efficacy and a higher rate of infusion reactions. In order to avoid the formation of these antibodies, low-dose methotrexate (usually 7.5 mg weekly) is frequently added to INX [1, 3]. (b) Etanercept (ETP) is a soluble receptor of human TNF. It is obtained by means of recombinant DNA technology, by fusion of the extracellular region of two type II TNF receptors and the Fc region of human immunoglobulin G1 [1, 3]. ETP binds both soluble TNF-*α* and TNF-*β*, leaving them biologically inactive. It is administered by subcutaneous route, once or twice a week. Skin reactions at the site of injection have been reported in up to 40% of cases, and although antibodies against ETP are present in less than 10%, a lower efficacy has not been observed [1, 3]. (c) Adalimumab (ADB) is a fully humanized IgG1 monoclonal antibody, specifically directed against TNF-*α*, which binds both its soluble and cell-bound forms. It is administered once every two weeks by subcutaneous injection. Due to its more recent release, data regarding safety, as well as the incidence of antibodies directed against ADB and their possible effect on its efficacy, are insufficient [3]. (d) Certolizumab pegol (CZP) is a pegylated humanized antibody Fab' fragment of a monoclonal antibody specifically directed against TNF-*α*, which binds both its soluble and the cell-bound forms. It is administered subcutaneously every other week [4]. (e) Golimumab (GLB) is a fully humanized IgG1 monoclonal antibody, specifically directed against TNF-*α*, which binds both its soluble and cell-bound forms. It is administered subcutaneously once a month [4, 5]. It should be administered in conjunction with methotrexate in rheumatoid arthritis (RA) [6].

These five TNF-blocking agents are currently licensed for the treatment of a variety of disorders, namely, RA (INX, ETP, ADB, CZP, and GLB), juvenile idiopathic arthritis (JIA) (ETP and ADB), ankylosing spondylitis (AS) (INX, ETP, ADB, and GLB), psoriasis (INX, ETP, and ADB), psoriatic arthritis (PsA) (INX, ETP, ADB, and GLB), Crohn's disease (CD) (INX, ADB, and CZP) and ulcerative colitis (UC) (INX and ADB) [3–5, 7–9]. Nonetheless, the TNF-blocking agents are being used in an increasing number of autoimmune disorders, in those cases which are severe and resistant, or intolerant, to standard immunosuppressive therapies. The aim of this paper is to discuss the available data regarding the off-label uses of the anti-TNF agents in three specific frequent disorders: Behçet's disease, sarcoidosis, and noninfectious uveitis. At present, there is no published literature with regard to the use of CZP in these three conditions, while that concerning the use of GLB is limited to a few cases of uveitis or retinal vasculitis with dissimilar results [5]. Therefore, we will focus on INX, ETP, and ADB.

## 2. Behcet's Disease

Behçet's disease (BD) is a systemic vasculitis involving arteries and veins of any size, with a chronic-relapsing course, of unknown cause and with HLA-B51 as an admitted predisposing factor. The main manifestation of BD is recurrent oral ulcers, to which genital ulcers and systemic manifestations may be associated. Thus, BD may also involve the eyes, skin, nervous system, joints, kidneys, and arteries and veins of all sizes. Since sensitive or specific laboratory tests or specific pathologic findings are currently absent, the diagnosis is based on clinical criteria. Topical treatment is initially used for oral ulcer and mild ocular involvement. In the rest of cases, other therapies have been employed, such as colchicine, steroids, dapsone, thalidomide, methotrexate, azathioprine, cyclosporine A, cyclophosphamide, and mycophenolate. Notably, the main issue is the lack of controlled evidence regarding therapeutic options, especially in cases of neurological, vascular, and gastrointestinal manifestations [10]. TNF-*α* is believed to be a central inflammation mediator in BD, and, consequently, the TNF-blocking agents have been used in this disorder with different results. 

As it will be detailed later on, existing evidence on anti-TNF therapy in BD suggests that INX seems to be effective in ocular inflammation (mainly in posterior uveitis with serious risk of view loss), as well as extraocular manifestations. On the contrary, ETP has apparently shown better results in mucocutaneous lesions, even though enough data regarding its efficacy in ocular and articular involvement are insufficient, chiefly based on a few single case reports [11–13]. In fact, Melikoglu et al., in a 4-week randomized, double-blind, placebo-controlled trial, observed that patients treated with ETP achieved sustained remission for oral ulcers and nodular lesions, whereas no significant differences could be found regarding genital ulcers and papulopustular lesions. Patients receiving ETP showed a lower number of arthritis episodes, although the difference was not significant [14]. Conversely, two patients with neuro-BD have been reported to respond to ETP [15, 16].

A panel expert meeting on BD held in May 2006 [17] recommended considering the use of INX in patients with two or more relapses of posterior uveitis or panuveitis per year, loss of visual acuity secondary to chronic macular cystoids edema, refractory parenchymal central nervous system disease, selected patients with intestinal inflammation, or in those with articular or mucocutaneous involvement which significantly affects their quality of life (in whom ETP might also be considered). In cases of bilateral posterior uveitis with serious risk of view loss, a single dose of INX could be administered in order to prevent irreversible damage of the retina with permanent visual acuity loss. After that, the usual immunosuppressive therapy would follow (cyclosporine A or azathioprine, or even interferon-alpha, combined with low-dose corticosteroid). Subsequent to the publication of these recommendations, several case series have been published regarding eye, central nervous system, and bowel involvement, which further support the role of INX in the treatment of BD, usually being well tolerated and with hardly any side effects (except for one case of cytomegalovirus colitis) [17–23]. INX has subsequently been approved in Japan for the use of BD-related uveoretinitis not responding to conventional treatments [17]. Before and after the aforementioned recommendations, several prospective studies on the therapeutic use of INX for posterior uveitis reported a sustained response, with improvement of visual acuity and reduction of eye inflammation, either complete (65%) or partial (24%). This ocular remission was better maintained if combination with immunosuppressive agents was employed (azathioprine, cyclosporine A, and/or methotrexate), although it was only statistically significant for combination with cyclosporine A [12]. In another prospective study, the authors found that the effects of INX on reducing ocular inflammation were significantly faster than those of intravenous methylprednisolone or intravitreal triamcinolone acetonide, while the effect on visual acuity did not differ among them [24]. Besides, the efficacy of INX on BD uveitis seems to be maintained in the long term [25]. On the other hand, the intravitreal use of a single dose of IFX has been tested on a 15-patient pilot study with BD-associated relapsing posterior uveitis at the beginning of a unilateral attack. The outcomes of this study were significantly positive, while no side effects were noticed, either ocular or extraocular [26]. However, despite the good results reported in all these studies, uveitis may recur. In fact, Yamada et al., in a retrospective study, observed that 13 of 23 patients presented with recurring uveitis following treatment with INX, with a variable timing. The authors gave three possible explanations: (1) it might be a rebound effect due to a quick discontinuation of the patients' previous immunosuppressants; (2) it might be the result of too long interval between infusions after the fourth one; (3) it might be the consequence of neutralization of INX by antibodies directed against it [27]. Furthermore, small prospective studies and some case series and isolated patients have shown excellent response to INX in gastrointestinal involvement, central nervous system manifestations, pulmonary aneurysms, and other vascular involvements but not in hepatic vein thrombosis [12, 28–30]. For example, in the prospective study by Iwata et al., 10 patients with severe gastrointestinal involvement and who were irresponsive or intolerant to corticosteroids responded rapidly and dramatically to INX monotherapy. Moreover, improvement of abdominal computed tomography and colonoscopy in the long-term evaluation was noted [31]. In another study, five patients with neuro-Behçet's disease and resistant to methotrexate and steroids received INX as add-on therapy. They all showed clinical amelioration, along with regression of parenchymal lesions in magnetic resonance imaging and decrease of IL-6 levels in cerebrospinal fluid [32]. However, not only has the clinical response of INX in BD been analyzed but also its effects on health-related and vision-related quality of life, with significant improvement of the scores [33].

As far as ADB is concerned, the available data were initially very limited. Nonetheless, since it shares a similar mechanism of action with INX, the results were therefore expected to be comparable to those of the latter. In fact, a good number of the cases reported in the literature refer to patients who were switched from INX to ADB due to intolerance or failure to treatment compliance in cases of eye involvement, with no loss of efficacy [34, 35]. A subsequent observational study reported either sustained remission or good response in 17 BD patients (including mucocutaneous and neurological manifestations, as well as retinal vasculitis), who had to be switched to ADB due to lack or loss of efficacy, or else, infusion reactions [36]. A more recent case series observed a clinical improvement following treatment with ADB in 17 out of 19 patients, mainly with ocular, gastrointestinal, mucocutaneous, and peripheral nervous system involvement. Five of them had previously received INX and 2 ETP, both of which had proved ineffective. Furthermore, the number and dose of previous immunosuppressants could be reduced. Only 1 patient had it stopped due to the development of urticaria-angioedema [37]. Further case reports or small case series have shown good results with ADB in patients with different manifestations (gastrointestinal, mucocutaneous, neurological, articular, ocular, and vascular), either in anti-TNF naïve patients or following failure of INX [38–40].

## 3. Sarcoidosis

Sarcoidosis is a chronic, multisystemic disorder of unknown etiology, whose main characteristic is the development of noncaseating granulomas. These lesions may involve any part of the body, mostly the lungs and the thoracic lymphatic nodes. Granulomas can also affect the nodes of other parts of the body, skin, and eyes. The diagnosis is based on both clinical and histopathological findings. Systemic steroids remain the mainstay of treatment. Other options include hydroxychloroquine, azathioprine, methotrexate, cyclophosphamide, and mycophenolate.

The fact of  TNF-*α* being one of the participating cytokines in the formation of the sarcoid granuloma, along with the successful use of immunomodulators inhibiting the TNF-*α* such as pentoxiphylline and thalidomide, has led to the utilization of anti-TNF agents in numerous patients. Most of them have received treatment with INX, and while skin involvement seems to show better results, those regarding pulmonary involvement tend to be less positive. Likewise, a good response to INX in other locations, such as ocular, neurologic, articular, cardiac, hepatic, renal, vertebral, and parotid gland, has also been reported [3, 38, 41–59]. A multicentre, double-blind, randomized, placebo-controlled trial on the efficacy of INX in 138 patients affected with extrapulmonary sarcoidosis was recently published. Severity of extrapulmonary sarcoidosis decreased in over 40% of patients compared to placebo after 24 weeks of treatment, even though this difference disappeared once INX was discontinued [60]. In addition, a retrospective study involving 54 patients with lupus pernio found that INX seemed to be superior to systemic steroids in achieving resolution or near resolution of lesions, with or without other additional medications [61]. A subsequent double-blind, placebo-controlled trial with 134 patients with chronic pulmonary sarcoidosis found a modest though significant increase of forced vital capacity following INX therapy. The clinical response was stronger in those patients with an important baseline systemic inflammatory profile (which included different chemokines, neutrophil-associated proteins, acute-phase proteins, and metabolism-associated proteins), and it correlated with the decrease of inflammatory serum proteins, namely, MIP-1*β* and TNF-RII [60]. Also, in a small case series, 9 patients with pulmonary involvement experienced an improvement in lung functions tests after treatment with INX, even though only one patient normalized chest radiograph [62]. Finally, two small, retrospective studies have assessed the long-term efficacy of INX in sarcoidosis with pulmonary and extrapulmonary involvement. In one of them, with 16 patients, 88% of patients maintained the good initial response on follow-up. Only one patient had INX discontinued due to adverse events [63]. In the other one, with 26 patients, a sustained remission or improvement in almost 59% of the organs evaluated was achieved. INX had to be withdrawn because of adverse events in 3 patients, namely, severe pneumonia, positive purified protein derivative tuberculosis skin test, and recurrent sinusitis [64].

On the contrary, results with ETP are discouraging, similarly to other granulomatous disorders [3, 42, 43]. In two small studies, one with patients with pulmonary sarcoidosis and another with patients with ocular sarcoidosis, ETP failed to provide any amelioration of the disease. In addition, the first trial had to be terminated due to excessive treatment failures [65, 66]. This lack of efficacy of  ETP in granulomatous conditions has been attributed to its different mechanism of action, compared to that of INX and ADB [56].

Experience with ADB, on the contrary, is more limited. Several case reports have shown ADB to be efficacious in patients with different involvements (systemic, cutaneous, lymphadenopathies, inner ear, neurological, pulmonary, ocular, vertebrae and bone marrow), in one of them after ETP failure [56, 59, 67–73]. More recently, a double-blind, placebo-controlled trial assessing the effect of ADB in 16 patients with cutaneous sarcoidosis was published. ADB proved to be effective in improving both clinical lesions (Physician Global Assessment, target lesion area and target lesion volume) and Dermatology Life Quality Index score, even though this effectiveness partially wore off after an 8-week period of ADB withdrawal [74]. Another recent prospective study with 26 patients with refractory posterior uveitis revealed improvement or stabilization of intraocular inflammatory signs in 85% and 15% of patients, respectively. Other indicator of disease activity (pulmonary lung tests, laboratory tests) also improved [75]. 

Finally, the report of the onset of new sarcoid granulomas following treatment with anti-TNF agents for another reason (RA, AS, PsA, and SAPHO syndromes) is but paradoxical. The locations affected vary (lungs, central nervous system, bone marrow, skin, eyes, lymph nodes, liver, kidneys, joints, and parotid gland), and all the three TNF-blocking agents have been associated with this unexpected adverse event. The sarcoid granulomas usually resolved upon cessation of  TNF-*α* blockade and/or increase or initiation of oral steroids [76–89]. Even more shocking are the cases reported in which sarcoid granulomas disappeared after switching from ETP to ADB (since the initial patient's conditions so required) or did not relapse after reintroducing the same agent [90, 91]. The mechanism by which anti-TNF agents could induce sarcoidosis remains unclear. On the one hand, the role of TNF-*α* in sarcoidosis seems to be partial and to change as the disease evolves [84]. On the other hand, there is evidence that TNF-*α* and interferon-*α* (IFN-*α*) show cross-regulation in vitro [92] and in vivo [93]. Thus, suppression of  TNF-*α* levels would lead to an increase in those of IFN-*α*. And IFN-*α* has been reported to induce sarcoidosis and other autoimmune diseases [94].

## 4. Noninfectious Uveitis

The results from experimental models in animals as well as studies in humans suggest that TNF may play a central role in promoting ocular inflammation. Thus, intravitreal injection of TNF in rabbits and Lewis rats has shown to induce the development of uveitis [95–97]. Likewise, increased levels of TNF have been detected in serum and/or aqueous humor of uveitis patients when compared with controls. Based on these observations, therapy with TNF blockade has been utilized in ocular inflammatory disorders, either isolated or associated with systemic conditions. Even if limited, the existing evidence implies that INX seems to be more effective than ETP in the treatment of ocular inflammation [42]. 

The reported cases and case-series regarding INX suggest its efficacy in treating noninfectious uveitis and other ocular inflammatory disorders. As far as uveitis associated with other disorders, such as JIA, AS, CD, psoriasis and Takayasu arteritis, is concerned, INX has revealed equally effective [7, 42, 55, 98–107]. Braun et al. [108] reviewed the outcomes of different open studies and placebo-controlled trials with AS patients treated with either INX or ETP. They found that attacks of anterior uveitis (AU) had become less frequent (15.6 per 100 patient-years in the placebo group versus 6.8 per 100 patient-years in the patients treated with anti-TNF agents [*P* = 0.01]). This reduction in frequency was more marked, though not significant, in the patients receiving INX than in those treated with ETP (3.4 per 100 patient-years and 7.9 per 100 patient-years, resp.). More recently, Cantini et al. observed that INX was effective and safe in the long term in 14 patients with idiopathic posterior uveitis [25]. Furthermore, Farvardin et al. treated 10 eyes of 7 patients with chronic persistent noninfectious uveitis with a single intravitreal injection of IFX, resulting in a significant improvement of visual acuity and reduction of central macular thickness [109]. Data regarding BD and sarcoidosis-associated uveitis have already been reviewed before.

As to ADB, it was initially mainly used in infantile noninfectious uveitis. Thus, Vazquez-Cobian et al. [110] treated 14 children with uveitis, 5 idiopathic, and 9 JIA associated. They obtained a reduction of inflammation in almost 81% of eyes. Additionally, ADB was found to be effective in 16 out of 18 children with uveitis treated by Biester et al. [111] (17 JIA associated and 1 idiopathic), and Gallagher et al. observed an inflammation decrease in 50% of the 10 eyes of the 5 patients receiving ADB [112]. Since ADB possesses a similar mechanism of action to that of INX, comparable results to those of the latter could be assumed. In fact, a retrospective study with 43 spondyloarthropathies treated with anti-TNF agents, noted that those receiving INX or ADB presented a lower rate of uveitis relapses than those treated with ETP (number of uveitis flares/100 patient-years before and during ETP treatment: 54.6 versus 58.5 [*P* = 0.92], number of uveitis flares/100 patient-years before and during INX or ADB treatment 50.6 versus 6.8 [*P* = 0.001]) [113]. A European multinational, open-label, nonplacebo-controlled trial with 1250 AS patients treated with ADB was recently published. In this study, it was observed that the AU attack rate was reduced by 51% in all patients, by 58% in the 274 patients with a previous history of AU, by 68% in the 106 patients with a recent history of AU, by a 50% in the 28 patients with active AU at the beginning of the trial, and by 45% in the 43 patients with chronic uveitis. Furthermore, AU attacks during ADB treatment were generally mild [114]. In another study by Díaz-Llopis et al., 19 patients with refractory autoimmune uveitis received treatment with ADB. After one year of follow-up, visual acuity had improved in 31% of eyes, control of intraocular inflammation had been achieved in 63% of patients, the cystoid macular edema present in 86% of eyes at baseline had disappeared in 55% of eyes, and all patients had been able to have their dosage of baseline immunosuppressants reduced at the end of follow-up in at least a 50%. Uveitis relapsed in 42%, but it was easily controlled with a single intraocular injection of steroids [115]. 

Further increasing evidence in recent years supports the benefits of ADB in the treatment of uveitis. Thus, a case interventional study with 17 children with chronic uveitis (9 of whom had previously received another anti-TNF blocker) found that ADB improved visual acuity. Ocular inflammation also improved or stabilized. However, it did not completely manage to avoid the need for steroid treatment. One patient had ADB discontinued due to the development of varicella zoster infection [116]. In a larger case series study (131 adults with idiopathic or secondary refractory uveitis), the authors observed a significant improvement of visual acuity as well as intraocular inflammation parameters after a 6-month period of treatment with ADB. Additionally, patients could significantly reduce their baseline immunosuppression load (8.81 [5.05] versus 5.40 [4.43]; *P* = 0.001). In fact, 85% of patients had been able to reduce at least 50% of their baseline immunosuppression load at the end of the study. Only 9 patients presented severe relapses during follow-up [117]. Three further case series with 31 (prospective), 60 (retrospective), and 21 patients (prospective), respectively, have shown equally positive outcomes when treating refractory uveitis with ADB [118–120]. 

Finally, two studies have compared ADB with INX in the treatment of uveitis. In one of them, 48 pediatric patients with JIA-associated AU from the National Italian Registry were treated with IFX, while 43 received ADB for the same reason. After at least one year of follow-up, remission rate was significantly higher with ADB (67%) than with IFX (43%) [121]. In the other one, an open-label prospective study with 33 children with chronic uveitis from different etiologies (16 received ADA and 17 INX), authors observed a significantly higher probability of remission with ADB than with INFX after 40 months of follow-up [122]. 

In contrast to these results, there exist multiple cases in the literature reporting the occurrence of uveitis following anti-TNF therapy. In this regard, a study based on a registry of uveitis cases developed under treatment with IFX, ADB, or ETP in the USA found a significantly higher number of cases related to the use of ETP than to the other two agents (43 with ETP, 14 with INX, and 2 with ADB), even when the patients whose underlying disorder was associated with uveitis had been excluded. The authors concluded that these results suggest a relationship with the development of agent-specific uveitis rather than with the anti-TNF blockers on the whole. Nonetheless, they admitted the lack of enough evidence to discourage the use of ETP in the treatment of uveitis [53]. In another subsequent study based on a French survey, 31 cases of new onset of uveitis during treatment with anti-TNF blockers were reported. Again, ETP was the agent most frequently associated with this event, 23 cases as opposed to 5 with INX and 3 with ADB. In addition, the author performed a review of the English literature, which produced comparable results: 121 cases (including those of the aforementioned study), 103 of which were on ETP at the time of apparition of the uveitis [123].

## 5. Safety Issues

No specific adverse effects of anti-TNF therapy when treating BD, sarcoidosis, and noninfectious uveitis have been reported, other than those already known to these agents. These adverse effects include infections (especially tuberculosis), demyelinating diseases, malignancies (lymphomas), allergic reactions, development of autoimmunity, hepatitis, and new onset or worsening of existing congestive heart failure. As to safety during pregnancy, anti-TNF agents are classified in Category B [60].

## 6. Conclusions

TNF blockade has widely been used off-label, even though there is not any trial-based evidence to support it, except for the experience provided by cases and case series. This experience, which is continuously increasing, has yielded encouraging results, especially regarding ocular, cutaneous, and articular involvement, both in the disorders for which this therapy is licensed and in those for which they are not, such as BD. As far as individual agents are concerned, the largest available experience and the best outcomes on the whole are with INX, particularly in ocular, neurological, and gastrointestinal involvement in BD, skin lesions in sarcoidosis, and noninfectious uveitis, associated or not to another disorder. Instead, ETP has not proved effective in granulomatous diseases, as it had already been observed in conditions for which the TNF blockade is approved, such as CD, which could be the consequence of a different composition and mechanism of action. As to ADB, the experience was initially very limited. Nowadays, however, the growing evidence suggests that ADB may be more effective than INX in certain cases, such as noninfectious uveitis. Besides, its subcutaneous administration, which allows the patient to self-administer it at home, along with a similar mechanism of action to that of INX, makes the future of ADB most promising. Finally, the safety profile of these agents needs to be more specifically established. Nonetheless, it seems clear from the published literature that incidence of opportunistic infections (mostly tuberculosis) is increased and so does the development of autoimmunity. On the contrary, aspects like the association with demyelinating diseases and the occurrence of lymphomas need additional clarification. In sum, further randomized, controlled trials are required to adequately assess the actual benefits and safety profile in the long term of anti-TNF agents in BD, sarcoidosis, and noninfectious uveitis.

## Figures and Tables

**Table 1:** Characteristics of the different anti-TNF agents.

	Infliximab	Etanercept	Adalimumab	Certolizumab pegol	Golimumab
Type	Monoclonal antibody against TNF-*α*	TNF soluble receptor	Monoclonal antibody against TNF-*α*	Monoclonal antibody against TNF-*α*	Monoclonal antibody against TNF-*α*

Composition	Chimeric antibody	Recombinant fusion protein	Recombinant monoclonal antibody	Monoclonal antibody	Monoclonal antibody

Origin	Human and murine	Human	Human	Human	Human

Mechanism of action	Binding to soluble and cell-bound TNF-*α*	Binding to soluble TNF-*α* and TNF-*β*	Binding to soluble and cell-bound TNF-*α*	Binding to soluble and cell-bound TNF-*α*	Binding to soluble and cell-bound TNF-*α*

Dose	3–5 mg/kg at weeks 0, 2, and 6; then, every 4–8 weeks	25–50 mg once or twice a week	40 mg every other week	400 mg every other week (maintenance every 4 weeks can be considered)	50 mg once monthly

Administration route	Intravenous	Subcutaneous	Subcutaneous	Subcutaneous	Subcutaneous
